# Tratamiento temprano ortodóncico/ortopédico en pacientes con anomalías sagitales de clase II. Una revision

**DOI:** 10.21142/2523-2754-1103-2023-165

**Published:** 2023-09-26

**Authors:** Christian Yánez-Zurita, Blanca Naranjo Freire, Arístides Martillo Chiriguaya

**Affiliations:** 1 División de Ortodoncia, Universidad Católica de Cuenca. Cuenca, Ecuador. chrisyanez1993@gmail.com Universidad Católica de Cuenca División de Ortodoncia Universidad Católica de Cuenca Cuenca Ecuador chrisyanez1993@gmail.com; 2 Facultad de Odontología, Universidad de Guayaquil. Guayaquil, Ecuador. valerianaranjo93@gmail.com, aris_rogel@hotmail.com Universidad de Guayaquil Facultad de Odontología Universidad de Guayaquil Guayaquil Ecuador valerianaranjo93@gmail.com aris_rogel@hotmail.com

**Keywords:** maloclusión Angle clase II, ortopedia, aparatos ortodóncicos funcionales, ortodoncia, Angle class II malocclusion, orthopedics, functional orthodontic appliances, orthodontics

## Abstract

**Introducción:**

Las anomalías sagitales de clase II tienen una prevalencia de entre el 18% y el 32% de la población. Para resolver este tipo de maloclusión, se han propuesto diversas terapias, algunas de las cuales implican aparatos ortodóncicos u ortopédicos funcionales. Sin embargo, sigue siendo un tema de discusión si los tratamientos deben iniciarse a temprana edad o si existen efectos adversos que pueden perjudicar estructuras del sistema estomatognático.

**Objetivo:**

Analizar los resultados obtenidos de un tratamiento temprano en pacientes con maloclusión sagital de Clase II mediante una revisión de literatura.

**Materiales y métodos:**

Se llevó a cabo una búsqueda avanzada con términos y conectores en las bases de datos Medline vía PubMed y Science Direct; se aplicaron criterios de inclusión y exclusión para la selección definitiva de los artículos incluidos.

**Resultados:**

A través de esta búsqueda se recopilaron, en total, 5909 artículos, de los cuales se consideraron 23 que cumplían con los criterios establecidos en el presente trabajo de revisión.

**Conclusiones:**

Existen dispositivos ortodónticos/ortopédicos encaminados a solucionar las características propias de las maloclusiones sagitales de clase II, pero antes de planificar un tratamiento temprano es fundamental realizar un diagnóstico certero para evaluar el tipo específico de aparatología que se requiere.

## Abstract (en)

**Introduction:**

Class II sagittal anomalies have a prevalence of between 18-32% of the population. To resolve this type of malocclusion, various therapies have been proposed, some of which involve functional orthodontic or orthopedic appliances. However, it is still a matter of discussion whether the treatments should be started at an early age in patients, or if there are adverse effects that could harm structures of the stomatognathic system.

**Objective:**

To analyze the results obtained from early treatment in patients with Class II sagittal malocclusion through a literature review.

**Materials and methods:**

An advanced search with terms and connectors was carried out in the Medline via Pubmed and Science Direct databases. Inclusion and exclusion criteria were applied for the definitive selection.

**Results:**

Through this search, a total of 5909 articles were collected, of which 23 were considered to meet the criteria established in this review work.

**Conclusions:**

There are orthodontic/orthopedic devices aimed at solving the characteristics of class II sagittal malocclusions, but before planning early treatment it is essential to provide an accurate diagnosis to evaluate the specific type of appliance that is required.

## INTRODUCCIÓN

Uno de los problemas más comunes en la consulta ortodóntica son las anomalías sagitales de clase II, ya que su ocurrencia oscila entre el 18% y el 32% de la población (^1^). Los factores etiológicos de este tipo de maloclusiones son la protrusión maxilar y la retrusión mandibular, siendo esta última la causa más común ^2^.

La ortodoncia funcional con aparatos removibles ha sido una opción terapéutica para las anomalías sagitales de clase II ^3^. El tratamiento iniciado antes del pico de crecimiento puberal mejora de gran forma la respuesta esquelética; sin embargo, en ocasiones es necesario complementar la terapia funcional con un tratamiento posterior con aparatología fija ^4^. Se han diseñado diversas aparatologías removibles encaminadas a corregir los efectos dentoalveolares y esqueléticos de las anomalías sagitales clase II ^1^^,^^5^; sin embargo, los resultados del tratamiento dependen en gran medida de la cooperación del paciente ya que la apariencia del aparato puede ser desagradable en las interacciones sociales ^4^^,^^5^.

Actualmente, se puede contar con dispositivos funcionales fijos que tienen la ventaja de no depender de la cooperación del paciente; sin embargo, a menudo, después de esto, se requiere una segunda fase con aparatología fija (*brackets*) para completar el tratamiento ortodóntico ^2^. Comúnmente, la elección de un aparato funcional en el manejo de la maloclusión de Clase II dependerá del operador y de los factores de costo ^6^. Por eso, en general, el tratamiento temprano para la maloclusión de clase II aún es tema de discusión en la profesión y literatura ortodóntica ^4^. El propósito de esta revisión de literatura fue analizar los resultados obtenidos del tratamiento temprano en pacientes con maloclusión sagital de Clase II.

## MATERIALES Y MÉTODOS

La presente investigación es una revisión narrativa de la literatura que incluyó el análisis meticuloso de la información contenida en artículos científicos. 

Para esto, se llevó a cabo una búsqueda avanzada en el periodo agosto/2022-diciembre/2022, con términos y conectores en las bases de datos PubMed y Science Direct, los cuales se detallan en la Tabla 1.

**Tabla 1:** Términos y conectores de búsqueda avanzada con sus respectivos resultados obtenidos

Base de datos	Estrategia de búsqueda	Resultados
PubMed	(Malocclusion, Angle Class II) AND (rehabilitation)	26
	(Angle Class II) AND (early treatment)	6
	(Malocclusion, Angle Class II) AND (therapy)	925
	(Malocclusion, Angle Class II) AND (surgery)	422
Science Direct	(Malocclusion, Angle Class II) AND	26
	(Angle Class II) AND (early treatment)	0
	(Malocclusion, Angle Class II) AND (therapy)	4122
	(Malocclusion, Angle Class II) AND (surgery)	382
Total		5909

Criterios de inclusión

• Artículos publicados en los últimos 5 años.

• Artículos escritos en idioma español e inglés.

• Solo de tipo ensayo clínico, ensayo controlado aleatorizado y estudio prospectivo.

Criterios de exclusión

• Estudios realizados en animales.

• Artículos que no contienen información con respecto a los tratamientos tempranos de maloclusiones sagitales clase II.

**Figura 1:**
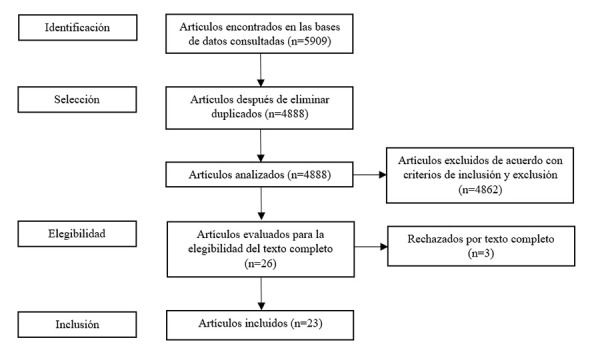
Diagrama de flujo PRISMA para inclusión de estudios

El proceso seguido desde la identificación hasta la selección de las fuentes bibliográficas empleadas en la investigación se detalla en el diagrama de flujo de PRISMA expuesto en la Figura 1.

## RESULTADOS Y DISCUSIÓN

Se realizó una búsqueda electrónica de publicaciones científicas realizadas en los últimos 5 años en las bases de datos PubMed y Science Direct. A través de esta búsqueda, se recopilaron en total 5909 artículos, de los cuales, tras aplicar los criterios de inclusión, exclusión y parámetros que consideraron apropiados los autores, se seleccionaron 23 para la revisión. Sus aspectos más relevantes se detallan en la Tabla 2.

**Tabla 2:** Características de los datos obtenidos de los artículos incluidos en la síntesis

Autor/año (país)	Titulo	Objetivo	Tipo de estudio	Edad promedio	Población de muestra / Aleatorización	Resultados	Conclusión
Abdelhady *et al*., 2020 (Egipto)	Maxillary molar distalization in treatment of angle class II malocclusion growing patients: Uncontrolled clinical trial	Evaluar los efectos del tratamiento de eficacia clínica de una técnica bucal simple para la distalización de los molares superiores (U6) utilizando un AE bucal directo	ECA	11 a 14 años	11 pacientes con relación molar MCII bilateral	Los dientes 1.6 y .26 fueron distalizados con una tasa de 0,30 mm y cantidad de distalización de 0,92 mm. No hubo pérdida de anclaje por el movimiento distal de todos los U6.	La distalización de los molares superiores mediante resorte cerrado y MN bucal es una técnica eficaz en un tiempo relativamente corto.
Al-Dumaini *et al*., 2018 (Siria)	A novel approach for treatment of skeletal Class II malocclusion: Miniplates-based skeletal anchorage	Evaluar el efecto de un nuevo enfoque (AE basado en MP bimaxilares) en el tratamiento de la MCII esqueletal en comparación con sujetos no tratados	EP	10 a 13 años	52 pacientes con MCII esquelética por retrusión mandibular. Se dividió en 2 grupos: grupo tratados con 4 MP bilaterales y grupo de control no tratados.	La longitud mandibular aumentó (3 mm) y la mandíbula se movió hacia adelante. Se encontró que la corrección del *overjet* (-4,26 mm) era un resultado neto de los cambios esqueléticos.	La nueva técnica, el AE basado en MP bimaxilares, es un método eficaz para el tratamiento de pacientes con MCII esqueletal a través de cambios esqueléticos evidentes, pero dentoalveolares mínimos.
Campbell *et al*., 2020 (Irlanda)	Frankl 2 appliance versus the Modified Twin Block appliance for Phase 1 treatment of Class II division 1 malocclusion in children and adolescents: A randomized clinical trial	Comparar el tratamiento de Fase 1, utilizando Frankl 2 o TB modificado, para la MCII división 1 en niños y adolescentes	ECA	11 a 14 años	60 pacientes con MCII división 1 Grupo FR2 (tratados con Frankl 2); Grupo MTB (tratados con TB modificado)	No hubo diferencias significativas entre ambos grupos con respecto a la duración del tratamiento, el número de roturas de aparatos y las perspectivas de los pacientes y padres.	La duración del tratamiento de la fase 1, el número de roturas del aparato, el resultado oclusal y las perspectivas del paciente y de los padres fueron similares en los tratamientos con el aparato Frankl 2 o TB.
Ciavarella *et al*., 2017 (Italia)	Treatment of hyperdivergent growth pattern and anterior open bite with posterior metallic bite planes	Evaluar los posibles efectos dentales y esqueléticos del aparato de intercepción de contacto oclusal de deglución en tratamientos de MCII hiperdivergente con evaluación cefalométrica utilizando un plano de referencia estable	ECA	26 pacientes en edades 9,46 ± 1,60 años	Todos los pacientes utilizaron el aparato durante 16 horas al día durante 24 meses, excepto en la mañana, para alimentarse y la higiene bucal.	Después de 24 meses de tratamiento, los autores observaron una modificación del crecimiento maxilar con una reducción de la divergencia con un aumento de la altura facial posterior, una modificación de la inclinación condilar y posición anterior del hioides. No se observaron modificaciones en el ángulo ANB. Tras el tratamiento se resolvió la mordida abierta con reducción de la inclinación de los U1.	El aparato de intercepción de contacto oclusal de deglución es fiable en pacientes en edad de crecimiento con un patrón de crecimiento hiperdivergente, mordida abierta anterior y MCII.
Cirgic *et al*., 2018 (Suecia)	A cost-minimization analysis of large *overjet* reduction with two removable functional appliances based on a randomized controlled trial	Evaluar y relacionar los costos sociales de reducir el *overjet* grande con DFP o AA, utilizando AMC	ECA	10,3 años	97 sujetos con MCII, división 1 con resalte de ≥ 6 mm divididos en 2 grupos: Grupo DFP y Grupo AA	Los costos directos y sociales fueron más bajos para el grupo DFP que para el grupo AA. El número de visitas fue menor en el grupo DFP.	La tasa de éxito de ambos aparatos fue baja. Sin embargo, DFP fue el enfoque preferido para la reducción de un resalte grande en la dentición mixta, ya que minimizó los costos y no hubo diferencias en los resultados clínicos entre DFP y AA.
DiBiase *et al*., 2020 (Reino Unido)	Post-treatment cephalometric changes in adolescent patients with Class II malocclusion treated using two different functional appliance systems for an extended time period: a randomized clinical trial	Evaluar el tratamiento y los cambios posteriores al tratamiento en pacientes que fueron tratados con aparatos funcionales TB o Dynamax durante un período prolongado de 15 meses	ECA	11 a 14 años	100 pacientes Grupo TB, y Grupo Dynamax	El avance mandibular fue de 3,5 mm (15 meses) y 0,3 mm (30 meses) en el grupo TB, y de 1,7 mm (15 meses) y 0,9 mm (30 meses) en el grupo Dynamax. La longitud mandibular aumentó 6,3 mm (15 meses) y 0,5 mm (30 meses) en el grupo TB, y de 4,0 mm (15 meses) y 1,5 mm (30 meses) en el grupo Dynamax.	El tratamiento resultó en un mayor crecimiento mandibular con TB que con Dynamax. En el período de seguimiento, hubo menos crecimiento en el grupo TB en comparación con el Dynamax.
Eissa *et al*. 2017 (Egipto)	Treatment outcomes of Class II malocclusion cases treated with miniscrew-anchored Forsus Fatigue Resistant Device: A randomized controlled trial	Evaluar los efectos sobre el esqueleto, dientes y tejidos blandos del FFRD utilizado con AE de MT y compararlos con los del FFRD convencional	ECA	11 a 13 años	38 pacientes con MCII Grupo 1 (tratados FFRD); Grupo 2 (tratados con FFRD y AE con MT); y Grupo de control	No se encontraron diferencias significativas entre los grupos de tratamiento.	El uso de MT con FFRD no mejoró el crecimiento mandibular hacia adelante ni evitó la inclinación labial de los L1.
El-Dawlatly *et al*., 2021 (Egipto)	The efficiency of mandibular mini implants in reducing adverse effects of class II elastics in adolescent female patients: a single blinded, randomized controlled trial	Limitar los efectos adversos de los elásticos de clase II mediante el uso de MT colocados en el arco mandibular en pacientes adolescentes de sexo femenino MCII	ECASC	13 a 17 años	28 pacientes femeninas con MCII, divididas en dos grupos: Grupo de intervención (tratados con MT) y Grupo convencional (terapia regular con elásticos clase II)	El cambio en la proinclinación de L1 no mostró diferencia entre los dos grupos, pero sí en la retroinclinación U1 y movimiento distal U1.	Los MT en conjunto con elásticos clase II no tuvieron efecto esquelético, principalmente dentoalveolar y no impidieron la proinclinación de los L1. Hubo más movimiento distal en los U1 en el grupo de anclaje esquelético que ayudó a mejorar el camuflaje de la MCII.
Elkordy *et al*., 2019 (Egipto)	Evaluation of the miniplate-anchored Forsus Fatigue Resistant Device in skeletal Class II growing subjects: A randomized controlled trial	Evaluar el uso del anclaje directo de MP junto con el FFRD en el tratamiento de la MCII esqueletal	ECA	11 a 13 años	48 pacientes mujeres con MCII esqueletal; se dividieron en dos grupos: Grupo FMP (tratados con Forsus más miniplacas); Grupo FFRD (tratados solo con Forsus); y Grupo de control (no tratados)	La longitud mandibular efectiva aumentó solo en el grupo FMP. Los L1 mostraron una proinclinación en el grupo FFRD y una retroinclinación en el grupo FMP. Se informó que la tasa de falla de las MP fue del 13,3%.	El uso de MP con el FFRD logró aumentar la longitud mandibular efectiva en sujetos con MCII a corto plazo. El FFRD anclado en MP eliminó la proinclinación desfavorable de los incisivos mandibulares en contraste con el FFRD convencional.
Golfeshan *et al*., 2018 (Irán)	Comparison between Classic Twin-block and a Modified Clear Twin-block in Class II, Division 1 Malocclusions: A Randomized Clinical Trial	Comparar los efectos dentoesqueléticos y la satisfacción del paciente con Clear TB modificado y un TB clásico	ECA	7 a 11 años	62 pacientes con MCII división 1 con mandibular retrognatia y un resalte mínimo de 4 mm, divididos en 2 grupos: Grupo Clear TG y Grupo Clásico TB	Ambos grupos mostraron avance mandibular, aumento de la proinclinación de los L1 y reducción de la sobremordida con una reducción significativamente mayor en el grupo clásico.	El aumento de la proinclinación de los L1 fue menor en el grupo Clear que en el clásico. La reducción de la sobremordida fue mayor en el grupo clásico que en el Clear.
Heino *et a*l., 2018 (Finlandia)	Effect of cervical headgear on dental arch area, shape and interarch dimensions: A randomized study	Estudiar los efectos del tratamiento temprano con un AC sobre el área del arco dental maxilar y mandibular, la forma y las dimensiones entre arcos	ECA	7,6 años	67 pacientes con MCII Divididos en 2 grupos: Grupo 1 (tratados con AC temprano durante 2 años) y Grupos de control (no tratados)	El AC aumentó el área del arco dental, las dimensiones sagitales en la línea sagital media y las dimensiones transversales en ambos arcos dentales en el grupo tratado.	El AC es un dispositivo de tratamiento eficaz para ganar espacio en ambas arcadas dentarias. Además, cuando se utiliza como tratamiento de fase temprana, la recaída es relativamente pequeña en comparación con el espacio ganado.
Idris *et al*., 2019 (Siria)	Soft- and hard-tissue changes following treatment of Class II division 1 malocclusion with Activator versus Trainer: a randomized controlled trial	Evaluar los cambios en los tejidos blandos y duros después de 12 meses de tratamiento en MCII división 1 en pacientes en crecimiento con un aparato funcional convencional (Activador modificado) versus un sistema entrenador miofuncional (T4K ®)	ECA	8 a 12 años	70 niños con MCII división 1 Divididos en 2 grupos: Grupo Activador y Grupo T4K	La mejora esquelética y dentofacial de MCII fue mayor en el grupo Activador: disminución del ángulo esquelético ANB, aumento mayor en el ángulo de convexidad facial y una reducción del resalte.	Los resultados del estudio actual indicaron que Activador fue más efectivo que T4K® en el tratamiento de pacientes en crecimiento de MCII división 1.
Julku *et al*., 2019 (Finlandia)	Dental arch effects after early and later timed cervical headgear treatment-a randomized controlled trial	Examinar las dimensiones de los arcos dentales en niños con MCII sin rotación mandibular posterior, según el momento del tratamiento de Arnés Cervical tipo Kloehn	ECA	7,2 años	67 pacientes con una MCII Divididos en 2 grupos: Grupo 1 (temprano, 26 meses) y Grupo 2 (tardío, 24 meses)	La longitud del arco maxilar y mandibular, así como los cambios transversales entre los caninos superiores y los primeros molares superiores aumentaron significativamente en hombres del Grupo 1.	El sexo masculino es el que más se beneficia de la sincronización temprana del tratamiento con AC, pues muestra mayores dimensiones al final del seguimiento. Los resultados indicaron una arcada dental superior más ancha y larga, y una expansión espontánea de la arcada dental inferior.
Julku *et al*., 2019 (Finlandia)	Comparison of effects of cervical headgear treatment on skeletal facial changes when the treatment time is altered: a randomized controlled trial	Evaluar los cambios faciales esqueléticos, particularmente en las dimensiones verticales, después del tratamiento con arnés cervical tipo Kloehn en niños cuando se altera el momento del tratamiento	ECA	7,2 años	67 pacientes con una MCII divididos en 2 grupos: Grupo 1 (tratamiento iniciado a la edad de 7,8 años) y Grupo 2 (tratamiento iniciado a la edad de 9,5 años)	La altura de la cara superior aumentó y la relación mandibular anteroposterior mejoró en ambos grupos. El ángulo gonial disminuyó significativamente en el grupo 1.	Aunque la altura de la cara superior aumentó, la mandíbula mostró rotación anterior después del tratamiento. Las diferencias encontradas entre los grupos de tratamiento temprano y tardío no fueron clínicamente importantes cuando se consideraron los resultados cefalométricos.
Kallunki *et al*., 2021 (Suecia)	Early headgear activator treatment of Class II malocclusion with excessive *overjet*: a randomized controlled trial	Comparar el tratamiento *headgear activador* para la MCII con *overjet* excesivo con sujetos de control no tratados	ECA	9,5 años	60 pacientes con overjet ≥ 6 mm Grupo tratamiento temprano *headgear activador*; Grupo sin tratamiento, el tratamiento se pospuso durante 2 años	El tratamiento temprano redujo significativamente el resalte y mejoró la relación molar. Los efectos se debieron principalmente a cambios dentoalveolares.	El principal efecto del tratamiento temprano con *headgear activador* para MCII con resalte excesivo es la reducción del resalte.
Kallunki *et al*., 2022 (Suecia)	Comparisons of costs and treatment effects-an RCT on headgear activator treatment of excessive *overjet* in the mixed and late mixed dentition	Comparar los costos y los efectos del tratamiento con headgear activador para la MCII con resalte excesivo entre los tratamientos iniciados en la dentición mixta y mixta tardía	ECA	8 a 10 años	56 niños con overjet ≥ 6 mm Grupo dentición mixta (tratamiento con *headgear activador* a partir de los 9 años); Grupo dentición mixta tardía (tratamiento con *headgear activador* a partir de los 11 años)	Los efectos del tratamiento en ambos grupos fueron la reducción del resalte y la mejora de la relación molar. No se encontraron diferencias con respecto a los costos de los tratamientos exitosos.	En cuanto a los costos y los efectos del tratamiento, no hay diferencia si el tratamiento del *overjet* excesivo con *headgear activador* se inicia en dentición mixta o dentición mixta tardía.
Lione *et al*., 2017 (Albania)	Mandibular response after rapid maxillary expansion in class II growing patients: a pilot randomized controlled trial	Evaluar la respuesta mandibular sagital inducida por la terapia de RME en pacientes con dentición mixta con MCII, comparando los efectos de la RME consolidado y la RME en bandas con un grupo de control de MCII sin tratar	EPAC	7 a 9 años	30 pacientes con MCII Grupo 1 (tratado con RME consolidado); Grupo 2 (tratado con RME en bandas); Grupo 3 (grupo de control sin tratamiento)	RME fue eficaz en la corrección de la deficiencia maxilar. No hubo mejora significativa de la relación anteroposterior del maxilar y la mandíbula a nivel esquelético y oclusal en ambos grupos. La férula acrílica RME tuvo efectos en la reducción de la dimensión vertical esquelética.	La expansión ortopédica no afectó la relación sagital de los pacientes MCII tratados en dentición mixta temprana en comparación con el grupo control no tratado.
Nagrik *et al*., 2020 (India)	A randomized clinical trial to assess the sagittal effects of TTA and NPE on skeletal class II malocclusion in growing patients during retention phase - A cephalometric study using a historical control group	Evaluar y comparar los cambios esqueléticos durante el período de retención después de la expansión con TTA y NPE en sujetos en crecimiento con MCII división 1, y comparar estos cambios con un control histórico emparejado	ECA	30 sujetos en edades entre 9-13 años con MCII división 1	15 sujetos fueron seleccionados para la expansión con TTA y los 15 restantes para expansión con NTE; además, se estableció un grupo control de similares características demográficas de un estudio previo, quienes nunca habían recibido tratamiento ortodóncico u ortopédico.	Después de 10 meses, todas las variables, excepto el ángulo SNA, mostraban diferencias significativas: ángulos SNB, ANB, evaluación de Witts y N perpendicular a pogonion, fueron mayores en el grupo de TTA que en los otros dos grupos.	Se produjo un avance sagital de la mandíbula cefalométricamente significativo después de la expansión con TTA y NPE en comparación con el control no tratado. TTA parece ser más eficiente para los cambios posicionales sagitales que el NPE.
Namera *et al*., 2020 (Siria)	Effects of low-intensity pulsed ultrasound applied on the temporomandibular joint region on the functional treatment of class II malocclusion: A randomized controlled trial	Evaluar los cambios dentoesqueléticos producidos por la combinación de la terapia LIPUS y el tratamiento funcional durante la corrección de la MCII esquelética	ECA	10,5 a 14 años	45 pacientes Grupo LIPUS (tratado con un aparato TB y terapia LIPUS); Grupo TB (tratado únicamente con aparato TB); y Grupo control	Se apreció una mejoría mayor en la longitud y posición mandibular en el grupo LIPUS que en el grupo TB.	La aplicación de la terapia LIPUS en combinación con el tratamiento funcional puede tener un gran efecto en la estimulación del crecimiento durante la corrección de MCII.
Pérignon *et al*., 2021 (Francia)	Effect of 970 nm low-level laser therapy on orthodontic tooth movement during Class II intermaxillary elastics treatment: a Randomized Clinical Trial	Evaluar el efecto de la terapia con láser de bajo nivel en el movimiento de los dientes durante el tratamiento con elásticos intermaxilares de Clase II	ECA	10 a 18 años	42 pacientes con MCII divididos en 2 grupos: Grupo A (tratados con láser activo: lado derecho; placebo: lado izquierdo), Grupo B (tratados con láser activo: lado izquierdo; placebo: lado derecho)	El tiempo para alcanzar la oclusión de Clase I en el grupo de láser activo (2,46 ± 2,1 meses) no fue significativamente diferente al del grupo de placebo (2,48 ± 2,0 meses). La distancia total de movimiento en el lado del láser activo (2,27 ± 1,5 mm) fue mayor que en el lado del placebo (1,64 mm)	No se pudo concluir que el uso de un láser de diodo reduce el tiempo para obtener el estado de Clase I en pacientes que usan elásticos de Clase II durante el tratamiento de ortodoncia. Sin embargo, se observó un impacto significativo en la aceleración del movimiento dental en el lado irradiado.
Talvitie *et al*., 2021 (Finlandia)	The impact of force magnitude on the first and second maxillary molars in cervical headgear therapy	Estudiar el efecto de la magnitud de la fuerza en U6 y U7 en la terapia con AC	ECA	9,7 años	40 pacientes divididos en 2 grupos: Grupo L (tratados con AC de magnitud ligera) y Grupo H (tratados con AC de magnitud fuerte)	La inclinación distal fue mayor en el grupo H. La inclinación se produjo en dirección distal durante la terapia en el grupo H en los U7 en comparación con la línea media o la línea condilar.	Con una gran magnitud de fuerza, U6 y U7 pueden inclinarse más fácilmente en la dirección distal incluso si se usa menos el CHG. La inclinación distal del molar puede considerarse un efecto secundario de la terapia con AC.
Telatar y Gungor, 2021 (Turquía)	Effectiveness of vibrational forces on orthodontic treatment: A randomized, controlled clinical trial	Evaluar la aplicación de fuerzas vibratorias en la tasa de distalización canina	ECA	13 a 18 años	20 pacientes con apiñamiento severo e indicación de exodoncia de primeros premolares para el tratamiento, divididos en 2 grupos: grupo de control y grupo de estudio	Las tasas de movimiento dental fueron de 1,06 mm/mes para los caninos superiores e inferiores en el grupo de control; y, en el grupo de estudio, 1,24 mm/mes para los caninos superiores y de 1,09 mm/mes para los caninos inferiores.	AcceleDent® Aura es un dispositivo fácil de usar; sin embargo, en el estudio, su aplicación no tuvo efectos positivos en las tasas de retracción canina.
Zhang *et al*., 2020 (China)	Effects of Twin-block vs sagittal-guidance Twin-block appliance on alveolar bone around mandibular incisors in growing patients with Class II Division 1 malocclusion	Comparar los efectos del aparato TB y el aparato TBGS en el hueso alveolar alrededor de los L1 en pacientes en crecimiento con MCII División 1, usando TCHC	ECA	11 a 13 años	25 pacientes Grupo TB; Grupo TBGS	En ambos grupos, la altura y el volumen del hueso alveolar labial de los L1 disminuyó después del tratamiento; los L1 se inclinaron y se desplazaron hacia el lado labial. Estos resultados fueron en menor proporción en el grupo TBGS.	Se observó pérdida de hueso alveolar labial alrededor de los L1 después del tratamiento con ambos tipos de aparatos en pacientes en crecimiento con MCII División 1.

U6: Primer molar maxilar. U7: Segundo molar maxilar. ECA: Ensayo clínico aleatorizado. MCII: Maloclusión de Clase II. MN: Minitornillo. AE: Anclaje esqueletal. MP: Miniplacas. TCHC: Tomografía computarizada de haz cónico. TB: *Twin-block*. TBGS: *Twin-block* de guía sagital. L1: Incisivos mandibulares. AC: Arnés cervical. LIPUS: Lo*w-intensity pulsed ultrasound.* TTA: *TransForce transverse appliance.* NPE: NiTi *palatal expander.* RME: *Rapid maxillary expansion*. EPAC: Ensayo piloto aleatorizado y controlado. FFRD: Forsus Fatigue Resistant Device. ECASC: Ensayo controlado aleatorizado simple ciego. AMC: Análisis de minimización de costos. AA: Activador de Andresen ligeramente modificado. DFP: Dispositivo funcional prefabricado. EP: Estudio prospectivo.

**Figura 2:**
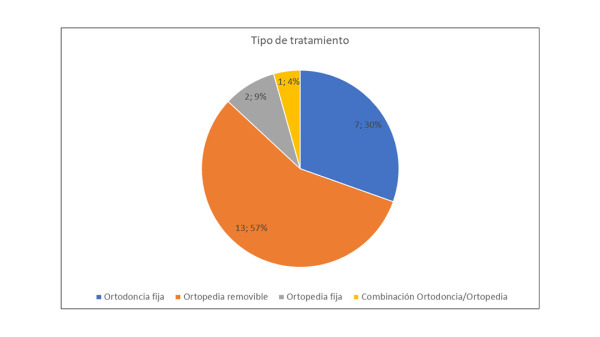
Diagrama del número y porcentaje de los artículos incluidos según el tipo de tratamiento que analizaron

De estos 23 artículos seleccionados e incluidos en la presente revisión de la literatura, en 13 (57%) los objetivos fueron evaluar tratamientos ortopédicos removibles, en 2 (9%) se evaluaron tratamientos ortopédicos fijos, en 7 (30%) se consideraron mecánicas de tratamientos ortodóncicos con aparatología fija y solo 1 (4%) tuvo como terapia una técnica combinada ortodóntica/ortopédica, tal como se detalla en la Figura 2.

Leiona *et al*. (2017), en su ensayo controlado aleatorizado, evaluaron la expansión mandibular rápida utilizando un dispositivo con acrílico y banda, el cual resultó eficaz en la corrección de la deficiencia maxilar, pero no mostró mejora en la relación anterosuperior de los maxilares ^7^.

Más tarde, autores como Golfeshan *et al*. ^5^, Zhang *et al*. ^8^ y DiBiase *et al*. ^9^ indicaron que el aparato *twin-block* muestra resultados eficientes para el avance mandibular. A la vez, Nagrik *et al*. ^10^ mencionaron en su estudio que los aparatos transversos linguales o palatinos Transforce y expansor palatino NiTi son útiles para el avance sagital mandibular.

Ciavarella *et al*. ^11^ demostraron la eficacia del aparato SOCIA (Swallowing Occlusal Contact Intercept Appliance) para controlar el crecimiento vertical de la mandíbula, la expansión maxilar y la posición de la lengua, pero no para modificar el ángulo ANB; además, se observó una función efectiva en el crecimiento e inclinación del cóndilo.

Eissa *et al*. ^2^ demostraron que el uso de microtornillos con dispositivo Forsus no mejora el crecimiento mandibular hacia adelante ni evita la inclinación labial de los incisivos mandibulares. Resultados similares se mostraron en el estudio de El-Dawlatly *et al*. ^12^, donde no se obtuvieron resultados esqueléticos significativos en la utilización de estos dispositivos de anclaje esqueletal situados en la mandíbula en combinación con elásticos. Abdelhady *et al*. ^13^ indicaron que la distalización de los primeros molares maxilares mediante resorte cerrado (resorte helicoidal cerrado de NiTi) y micro tornillo bucal, es una técnica eficaz y dependiente del incumplimiento en un tiempo relativamente corto.

Al-Dumaini *et al*. ^14^, en su ensayo controlado aleatorizado, utilizaron un anclaje esquelético bimaxilar con mini placas que demostró eficacia en maloclusiones esqueléticas clase II como intervención temprana. Esto concuerda con el estudio de Elkordy *et al*. ^15^ quienes señalan que el uso de miniplacas junto con dispositivos Forsus es eficaz para el aumento mandibular a corto plazo al eliminar la proinclinación de los incisivos mandibulares.

Kallunki *et al.* ^(3, 4)^ mencionan que el arnés activador no solo es efectivo para la disminución del resalte excesivo, sino también en la corrección de la relación molar en los tratamientos iniciados en la dentición mixta y mixta tardía. Sin embargo, Cirgic *et al*. ^17^ hallaron una baja tasa de éxito para la corrección del *overjet* al utilizar el activador de Andresen y el aparato funcional prefabricado.

Julku *et al*. ^(18, 19)^ demostraron el beneficio del uso de arnés cervical en la expansión del arco dental superior y una expansión simultánea del arco inferior en maloclusiones clase II. Adicionalmente, indican que el tratamiento con arnés cervical produce un aumento de la altura facial superior en el tratamiento temprano o tardío. Esto concuerda con el estudio elaborado por Heino *et al*. ^20^, quienes señalan que, además de los efectos en las dimensiones sagitales, se observa un aumento del arco dental y dimensiones transversales en ambos arcos dentales. Talvitie *et al*. ^21^ mencionan que la aplicación de fuerzas moderada (500 g) produce una mayor inclinación distal de los primeros y segundos molares en el tratamiento con arnés cervical.

Namera *et al*. ^1^ revelaron que la aplicación de la terapia LIPUS (ultrasonido pulsado de baja intensidad), en combinación con el tratamiento funcional, tiene mejores efectos en la estimulación del crecimiento dentoesquelético mostrando una reducción en cuanto a la duración del tratamiento. Pérignon *et al*. ^22^ revelaron que la aplicación de terapia de láser de bajo nivel en el movimiento de los dientes durante el tratamiento con elásticos intermaxilares de Clase II no redujo el tiempo necesario para obtener una oclusión de Clase I, pero se observó una aceleración significativa en el movimiento dental. Telatar y Gungor ^23^ demostraron que la aplicación de fuerzas vibratorias para acelerar la distalización canina con aparato fijo no tuvo efecto positivo, ya que se obtuvo resultados similares con aquellos tratamientos donde no se aplicaron estas fuerzas.

Los resultados de este estudio muestran la eficiencia de las aparatologías para solucionar los problemas presentes en pacientes con maloclusiones de clase II. Dispositivos como *twin-block*, aparato transverso lingual o palatino Transforce, y expansor palatino NiTi, así como el anclaje esquelético con miniplacas, resultan ser eficaces para el avance mandibular. Por otro lado, el uso de aditamentos como resorte helicoidal cerrado y microtornillos bucales, así como uso de un activador modificado, arnés activado y arnés cervical son ideales para la corrección de la relación molar y la disminución del *overjet*. Sin embargo, realizar acciones de biomecánica en edades tempranas para solucionar las características propias de las maloclusiones sagitales de clase II no garantiza al 100% el éxito del tratamiento. Por esa razón, antes de planificar un tratamiento temprano con aparatología ortodóncica, ortopédica o una terapia combinada es fundamental otorgar un diagnóstico certero, para evitar posibles efectos indeseados que pueden afectar el equilibrio del sistema estomatognático.

## CONCLUSIONES

El tratamiento de las anomalías sagitales en edades tempranas es una alternativa frecuente para la corrección de maloclusiones Clase II, que permite la obtención de una óptima función en los estadios de desarrollo en la mayoría de casos, con la finalidad de que los cambios estructurales y funcionales se mantengan a lo largo de los años. 

## Fuente de financiamiento:

La presente investigación fue autofinanciada
